# Efficient Biodegradation of Patulin by *Aspergillus niger* FS10 and Metabolic Response of Degrading Strain

**DOI:** 10.3390/foods12020382

**Published:** 2023-01-13

**Authors:** Yang Yang, Jian Ji, Shang Wu, Yongli Ye, Lina Sheng, Yinzhi Zhang, Xiulan Sun

**Affiliations:** 1 School of Food Science, State Key Laboratory of Food Science and Technology, National Engineering Research Center for Functional Foods, School of Food Science Synergetic Innovation Center of Food Safety and Nutrition, Joint International Research Laboratory on Food Safety, Jiangnan University, Wuxi 214122, China; 2 College of Food Science and Pharmacy, Xinjiang Agricultural University, Ürümqi 830052, China

**Keywords:** patulin, *Aspergillus niger*, degradation, toxicity, metabolomics, apple pomace

## Abstract

Patulin, a mycotoxin commonly found in fruits and derived products, causes serious health problems for humans and animals worldwide. Several microbial strains have been observed to possess the ability to effectively remove patulin. However, these methods are presently associated with disadvantages such as low degradation efficiency and an unclear biodegradation mechanism. In the current study, the characteristics of patulin degradation via *Aspergillus niger* FS10 were evaluated, and the mechanisms involved were analyzed using metabolomics technologies. The results showed that the suspension of *A. niger* FS10 could degrade 94.72% of patulin within 36 h. The moment concentration pf patulin was 0.116 μg/mL, and the detection limit value was 0.01 μg/mL. In addition, the patulin content was reduced to levels below the detection limit within 48 h. *A. niger* FS10 mainly degrades patulin by producing intracellular enzymes, which can convert patulin into ascladiol. This degradation method can effectively reduce the damage caused by patulin to HepG2 cells. In addition, the patulin treatment significantly affects the pentose phosphate pathway and the glutathione pathway. These two metabolic pathways are speculated to be closely related to patulin degradation via *A. niger* FS10. The incubation of *A. niger* FS10 with patulin-contaminated apple pomace can not only eliminate patulin but also increase the utilization of apple pomace. Therefore, our research results provide a new method for addressing patulin contamination in the food and feed industries.

## 1. Introduction

Patulin, a toxic secondary metabolite from *Aspergillus*, *Byssochlamys*, and *Penicillium* [1], is found in various fruits, vegetables, and derived products [2]. Approximately 25% of the loss of food products in developed countries and 50% in developing countries is attributed to patulin contamination [3]. Patulin, a primary pollutant, has drawn global attention. In Michigan, the United States, 23% of fruit wine and fruit vinegar was contaminated with patulin, with a maximum content of 2.7 mg/L [4]. Patulin was also detected in 97.7% of concentrated apple juice in Shaanxi, China [5]. Apple pomace is a by-product of apple juice production. It is often used as animal feed; however, owing to its high water content, apple pomace is easily infected by microorganisms and produces large amounts of patulin, thus endangering the health of humans and animals. Patulin leads to various types of animal toxicity, including cytotoxicity [6], chronic toxicity [7], and acute toxicity [8]. Specifically, patulin presents a serious threat to the health of infants and young children [9]. Thus, many countries have formulated regulations to limit the maximum content of patulin in fruit and vegetable products [10].

The current methods of patulin degradation mainly include physical, chemical, and biological methods. However, the application of physical and chemical methods may lead to secondary pollution, nutrient loss, low removal efficiency, and high costs [11]. By contrast, biodegradation is considered a safe and environmentally friendly method [12]. *Rhodotorula mucilaginosa* [13], *Lactobacillus casei* YZU01 [14], and *Pichia caribbica* [15] can effectively degrade patulin. Dong et al. screened a yeast strain, *Kodameae ohmeri*, that can tolerate 100 μg/mL of patulin with a degradation rate reaching 93% at 35 °C [12]. Meng et al. isolated a new *Enterobacter cloacae* subspecies and proved that it could convert patulin into E-ascladiol under the action of ribonucleoside diphosphate reductase (*NrdA*) [16]. However, patulin degradation via *A.niger* is rarely reported.

*A. niger* is an important industrial strain and has been widely used in mycotoxin degradation in recent years. The *A. niger* strain isolated from coffee beans can completely inhibit the production of ochratoxin [17]. Xing et al. isolated 20 *A. niger* strains from peanuts; among these strains, it was determined that 14 can completely inhibit the production of aflatoxin B1 (AFB1) by co-cultivation [18]. Our previous study on the inhibition of AFB1 production and zearalenone (ZEN) degradation by *A. niger* achieved ideal results [19]. These studies indicate that *A. niger* may be a potential strain for mycotoxin degradation.

In the current study, the degradation of spore suspensions, mycelium, and culture filtrates by *A. niger* FS10 was analyzed to identify the main components of *A. niger* FS10 that degrade patulin. The degradation products of patulin were analyzed using Q Exactive Focus high-resolution liquid chromatography. A HepG2 cell model was used to evaluate the toxicity of the patulin degradation products. Metabonomics was used to investigate the metabolic response of *A. niger* FS10 to patulin degradation. Meanwhile, *A. niger* FS10’s degradational effects on patulin in apple pomace evaluated.

## 2. Materials and Methods

### 2.1. Fungal Strain and Culture Conditions

A food-grade and food-safe strain of *A. niger* FS10 was isolated from Chinese fermented soybean. The culture conditions used in this study were similar to the conditions in the previous study, with certain modifications. *A. niger* FS10 was cultured on Potato Dextrose Agar (Shanghai Bioway Technology Co., Ltd., Shanghai, China) at 28 °C for 3 d and, consequently, produced spores. Part of the spore volume was collected with 0.9% saline containing 0.05% Tween, and the spore concentration was adjusted to 10^6^ CFU/mL. Subsequently, 2% (*v*/*v*) spore suspension was added to the control Potato Dextrose Broth (PDB, Shanghai Bioway Technology Co., Ltd.) and the PDB containing 2 μg/mL of patulin. The mixture was then incubated at 180 rpm for different durations at 28 °C to obtain mycelia, sterile culture filtrates, and fungal suspensions of *A. niger* FS10.

### 2.2. Chemicals

Patulin (analytical standard, purity ≥98%) was purchased from Glpbio (Montclair, CA, USA). The ultrapure water used in this study was produced using the Millipore-Q SP Reagent Water system (Millipore, Bedford, MA, USA). Chromatography-grade ethyl acetate and ethanol were purchased from Chemical Reagent Co., Ltd. (Shanghai, China). Other chemicals were of analytical grade. All reagents were used without further purification.

### 2.3. Extraction and Determination of Residual Patulin

Residual patulin in 1 mL of the liquid culture was extracted 3 times with 3 mL of ethyl acetate, which was then evaporated with a vacuum freeze-dryer. The samples were subsequently redissolved with ultrapure water (pH = 4.0). The reconstituted solution was passed through an organic microporous membrane of 0.22 μm (Thermo Fisher, Waltham, MA, USA). The concentration of patulin was analyzed using the Agilent 1260 HPLC system (Agilent, Santa Clara, CA, USA). Then, patulin was separated on a Zorbax SB-C_18_ column (150 × 4.6 mm, 5 μm; Agilent, Santa Clara, CA, USA) with a mobile phase of ultrapure water (pH = 4.0) under the following conditions: ultrapure water:acetonitrile [90:10 (*v*/*v*)] at a flow rate of 0.8 mL/min; injection volume of 15 μL; and assay temperature of 25 °C. UV detection was performed at 276 nm.

### 2.4. Biodegradation Assay of Patulin

A spore suspension (1 × 10^6^ CFU/mL) from *A. niger* FS10 was inoculated into 50 mL of the PDB medium containing 2 μg/mL of patulin and cultured in an incubator (180 rpm; 28 °C). The concentration of patulin was measured every 4 h. In each group, three samples were formed in parallel biological replicates.

The degradational activity of the culture filtrate, mycelium, and spore was determined to further characterize the degradation of patulin. FS10 mycelia and culture supernatant were separated by vacuum filtration after incubation for 36 h in the PDB. The mycelia and culture filtrate were collected separately. The culture filtrate was filtered through the organic microporous membrane (0.22 μm), and the mycelia were washed with phosphate-buffered saline (PBS, pH = 7.3) (4 °C) three times. The germ-free filtrate was divided into two groups: Group I, with patulin at a final concentration of 2 μg/mL, and Group II, which was inactivated (100 °C; 10 min) and contained patulin at a final concentration of 2 μg/mL. The fungal mycelia were then divided into viable mycelia (untreated) or autoclaved mycelia (121 °C for 20 min). The mycelial pellets were washed with PBS (pH = 7.3) three times. Subsequently, 0.1, 0.3, 0.6, 0.8, and 1.0 g pellets were suspended in 9 mL of PBS solution infused with 1 mL of patulin with a concentration of 20 μg/mL and then incubated at 30 °C for 36 h at 200 rpm under aerobic conditions.

Harvested spores were washed with 0.01 M PBS three times and prepared in suspensions of 0, 1, 2, 5, 7, and 9 mL. The final volume was increased to 10 mL by adding PBS. Before patulin was added as described earlier, spore suspensions were determined using the optical density (OD) at 560 nm. All aforementioned experiments were prepared in three biologically parallel setups.

### 2.5. Q Exactive Focus Analysis for Degradation Products

Samples at each time point were detected using the Q Exactive Focus system (Agilent Technologies Inc., Santa Clara, CA, USA) following the method described by Xu et al. [20]. Data were then converted into the “.abf” format and analyzed by the MS DIAL software. Subsequently, MS and MS/MS data were imported in MS-FINDER for unknown identification.

### 2.6. Toxicity Evaluation of Patulin Degradation Products by the HepG2 Cell Model

HepG2 cells were cultured for continuous logarithmic-phase growth in an MEM medium with 10% FBS (Thermo Fisher, Waltham, MA, USA) in T-150 tissue culture flasks. The cells were incubated at 37 °C in a humidified atmosphere of 5% CO_2_ and 95% air. Freshness was maintained by changing the culture medium every other day or as needed.

Patulin (degraded by *A. niger* FS10 for 0 h) and degradation products (degraded by *A. niger* FS10 for 48 h) were extracted from the PDB by using ethyl acetate. The impurities were removed using a solid-phase purification column. The purified patulin and degradation products were freeze-dried in a vacuum. The products were then diluted with the MEM medium to obtain patulin, which was used as the treatment group; the degradation product, which was used as the product group; and MEM, which was used as the control group working solution.

Toxicity evaluation was conducted using a CCK-8 assay kit (Beyotime Biotechnology, Shanghai, China). The optical density of each well was measured using a microplate reader at 450 nm. The cell viability *(CV*) of the cells was calculated using Equation (1) as follows:

(1) $$CV=\frac{AS-AB}{AC-AB}\times 100\%$$

where *AS* is the experimental group, *AC* is the control group, and *AB* is the blank group. The apoptosis status was determined using the Annexin V–FITC/PI Apoptosis Detection Kit (Beyotime Biotechnology, Shanghai, China). The samples were monitored with an FACS Calibur flow cytometer (FL-1 530 nm, FL-2 585 nm) (Becton-Dickson, San Jose, CA, USA). Data were analyzed and presented using the FlowJo 10 software (BD, Franklin, NJ, USA).

### 2.7. Metabolite Analysis

The mycelia were collected into a 1.5 mL Eppendorf centrifuge tube and washed three times with 1 mL of PBS in an ice bath at 4 °C. Subsequently, 1 mL of the cold-quenching solvent (ice-cold methanol/H_2_O (3:2)) was added into each centrifuge tube and then shaken at 4 °C for 5 min at 9000× *g* to discard the supernatant. Into each test tube, 1 mL of the extraction solvent (acetonitrile/isopropanol/H_2_O (3:3:2, *v*/*v*)) was added. The mycelia were ground and crushed with GenGrinder at 1500 rpm for 30 s and 8 cycles. After centrifugation (4 °C for 10 min at 12,000 rpm/min), 950 μL of the supernatant was collected and divided evenly in two, with 475 μL per serving; these samples were used for analysis and backup. Amounts of 10 μL from each sample were taken for quality control. The samples were vacuum freeze-dried for analysis.Methoxyamine hydrochloride (MeOX) at 40 mg/mL was added to 10 μL of the dried sample and incubated at 30 °C for 90 min at 1200 rpm/min. Subsequently, 92 μL of N-methyl-N-(trimethylsilyl) trifluoroacetamide (MSTFA) was added to the centrifuge tube for derivatization and vortexed for 10 s. The centrifuge tube was placed in a metal bath at 37 °C and 1200 rpm/min for 30 min. Fatty acid methyl esters (FAMEs; C_8_–C_30_, dissolved in chloroform) were used as internal standards. Finally, the supernatant was used for computer analysis.

LECO Pagasus BT gas chromatography time-of-flight mass spectrometer (LECO Corporation, Lake County, CA, USA) was used to detect intracellular metabolites following the method described by Xu et al. [20].

### 2.8. Degradation of Patulin in Apple Pomace by A. niger FS10

Sterile water was added to the patulin-contaminated apple pomace at a ratio of 1:3 (g/mL), and the FS10 spore suspension was inoculated into the apple pomace. The mixture was then incubated at 28 °C for 4 d. The residual amount of patulin in the apple pomace was measured daily. Simultaneously, the concentrations of cellulose, protein, fat, and total amino acids in the apple pomace before and after fermentation were determined.

### 2.9. Statistical Analysis

Data were processed and analyzed using SPSS 26 (IBM, Armonk, New York, NY, USA), and images were generated using GraphPad Prism 8 (GraphPad Software, San Diego, CA, USA). The metabolomics software MS-DIAL (UC, Davis, CA, USA) was used to process the data, and the programming language R was used to visualize the data. MetaboAnalyst 5.0 (https://www.metaboanalyst.ca; accessed on 12 October 2022) was used for pathway enrichment analysis. Data visualization in R was conducted. The significance threshold was set at *p* < 0.05.

## 3. Results

### 3.1. Biodegradation Analysis of Patulin by Aspergillus niger FS10

**Figure 1:**
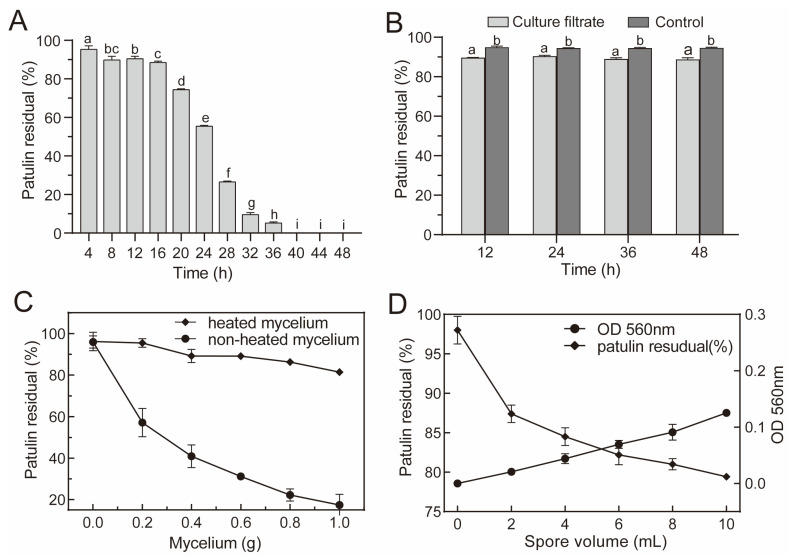
Biodegradation of patulin by *A. niger* FS10. (**A**) Efficacy of fungal suspension of *A. niger* FS10 with respect to the degradation of patulin; (**B**) efficacy of culture filtrate of *A. niger* FS10 with respect to the degradation of patulin; (**C**) Efficacy of mycelia of *A. niger* FS10 regarding the degradation of patulin; (**D**) efficacy of spores of *A. niger* FS10 towards the degradation of patulin. Each data bar indicates the means of three replicates ± standard deviation. Different letters indicate a significant difference between them (*p* < 0.05).

Patulin degradation via *A. niger* FS10 in the PDB was evaluated. The fungal suspension of *A. niger* FS10 is shown in Figure 1A; as the culture time was extended, the patulin content gradually decreased. The residual rate of patulin decreased rapidly from 95.35% to 5.29% from the initial period of 4 h to 36 h; after 40 h, the residual rate of patulin was 0%, which indicated that patulin had been completely degraded. To further determine its biodegradation characteristics, patulin was cultured with *A. niger* FS10 sterile fermentation broth, mycelium, and spores (Figure 1B–D). The residual patulin content in the *A. niger* FS10 sterile broth culture filtrate group was compared with that of the control group, and the concentration of patulin was reduced in the culture filtrate group. However, no significant difference was observed with respect to the removal of patulin by the culture filtrate with different culture durations. In Figure 1C, when the mycelium increases from 0 to 1.0 g, the residual rate of patulin in the untreated group decreased from 95.96% to 17.41%, and that in the heat-treated group decreased from 96.22% to 81.42%. The degradation rate of patulin by the mycelium in the untreated group increased by 64.01% relative to that of 1.0 g achieved by the mycelium in the heat-treated group. As shown in Figure 1D, as the volume of the spore suspension increased, the residual rate of patulin in each flask gradually decreased, indicating that the FS10 spores exert a certain reductive effect on patulin.

### 3.2. Biodegradation Product Analysis of Patulin by Aspergillus niger FS10

**Figure 2:**
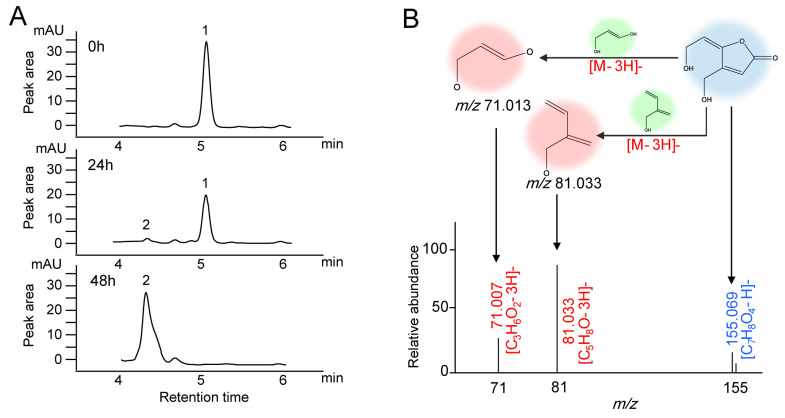
Chromatograms and mass spectra of patulin and suspected degradation products produced by *A. niger* FS10. (**A**) Time-course HPLC-UV of patulin removed by *A. niger* FS10 over 0 h, 24 h, and 48 h. (**B**) Mass spectra of suspected degradation products produced by *A. niger* FS10.

*A. niger* FS10 and patulin were co-cultured for biodegradation experiments, and analyses of the biodegradation products of patulin were performed using high-performance liquid chromatography and Q Exactive Focus. In Figure 2A, at 0 h, only one patulin peak (peak 1) was detected, and its retention time was 5.03 min. After incubation for 24 h, a new peak (peak 2) appeared with a retention time of 4.31 min. After the incubation time was extended to 48 h, the peak of patulin (peak 1) disappeared completely, and the increased peak area of the degradation product (peak 2) was consistent with the decreased peak area of the patulin peak (peak 1).

The degradation product was determined in accordance with the hydrogen rearrangement rules proposed by Tsugawa et al. [21]. The MS DIAL and MS-FINDER programs were used to analyze the MS and MS/MS characterization of the degradation sample from the 48 h time point. In Figure 2B, the peak of the degradation product with the ion is *m*/*z* 155.069 in the negative ionization mode and is identified as the [M−H]^−^ precursor. Two major fragmentation ions were found, *m*/*z* 71.013 and *m*/*z* 81.033, which were identified as [C_3_H_6_O_2_-3H]^−^ and [C_5_H_8_O-3H]^−^. These anions were derived from the collision-induced loss of 3H from the [M−H]^−^ ion of the degradation product. The MS/MS profile conforms to the collision fragmentation characteristics of ascladiol. Fungi have been reported to convert patulin to ascladiol, and the retention time of the ascladiol peak in the HPLC chromatogram was shorter than that of the patulin peak [22]. This finding is consistent with our research results.

### 3.3. Patulin Toxicity Reduction in the HepG2 Cell Model

**Figure 3:**
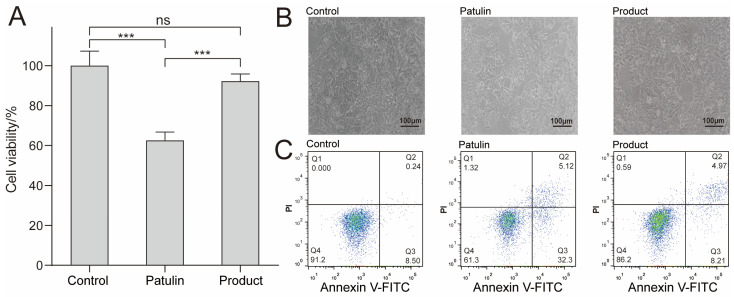
Effects of MEM (control group), patulin (treatment group), and patulin’s degradation products (product group) on the cytotoxicity and apoptosis of HepG2. (**A**) Cell viability measured by CCK8 method, where “***” means *p* < 0.001, and “ns” means no significant difference. (**B**) Cell morphology determined by an inverted microscope; the scale bar is 100 μm. (**C**) The effect of Patulin and its degradation products on cell apoptosis. After FITC/PI staining, flow cytometry was used to detect cell apoptosis. The flow cytometry chart indicating the percentages of surviving cells, early apoptotic cells, late apoptotic cells, and necrotic cells; at least three samples were analyzed for each condition.

To verify the effect of this degradation method on the reduction of patulin toxicity, the CCK8 method was used. The effects of patulin and its degradation products on the viability of HepG2 cells were evaluated. For this assay, HepG2 cells were exposed to patulin and the product at a concentration of 5 μg/mL for 24 h (Figure 3A), and the non-stimulant group served as the control group. The results indicated that the cell viability of the group exposed to patulin was 61.2%, whereas the cell viability of the group exposed to degradation products reached 91.5%. These results indicate that the toxicity of patulin was considerably reduced after degradation. An inverted microscope was used to further observe the cell morphology of the different experimental groups. As shown in Figure 3B, the group exposed to degradation products was similar to the control group, with strongly adhered HepG2 cells, a complete morphology, and high cell density. However, in the group exposed to patulin, the cells poorly adhered to the wall, many cells were detached from the wall, and the cell density was low. The cell flow diagram (Figure 3C) shows that the apoptotic rate of the control group was 8.74%, and the apoptotic rate of patulin was increased to 37.42%; notably, the apoptotic rate of the degradation products was reduced to 13.18% (*p* < 0.05). All the aforementioned results verify the low cytotoxicity of the degradation products.

### 3.4. Multivariate Statistical Analysis of the Metabolite Spectrum

We investigated the metabolic feedback of *A. niger* FS10 to patulin from the perspective of metabonomics. Principal component analysis (PCA) was used to preliminarily determine the similarities and differences between the control group and the patulin treatment groups with different incubation times (24, 36, and 48 h), and similar samples were clustered (Figure S1). Significant differences were determined via PCA between the control group and the patulin treatment group (Figure S1A (24 h) and Figure S1B (36 h)). Meanwhile, the control group and the patulin treatment group were clustered for 48 h, indicating that the metabolism profiles of the control group and the patulin treatment group showed consistency after patulin was completely degraded.

Partial least squares discriminant analysis (PLS-DA) is a powerful statistical modeling tool that can be used with nuclear magnetic resonance, mass spectrometry, or other analytical data for an in-depth study of individual experimental groups. As shown in Figures S2 and S3, this approach exhibits good reliability in predicting the permutation test model, rendering it suitable for the further screening of differential metabolites.

**Figure 4:**
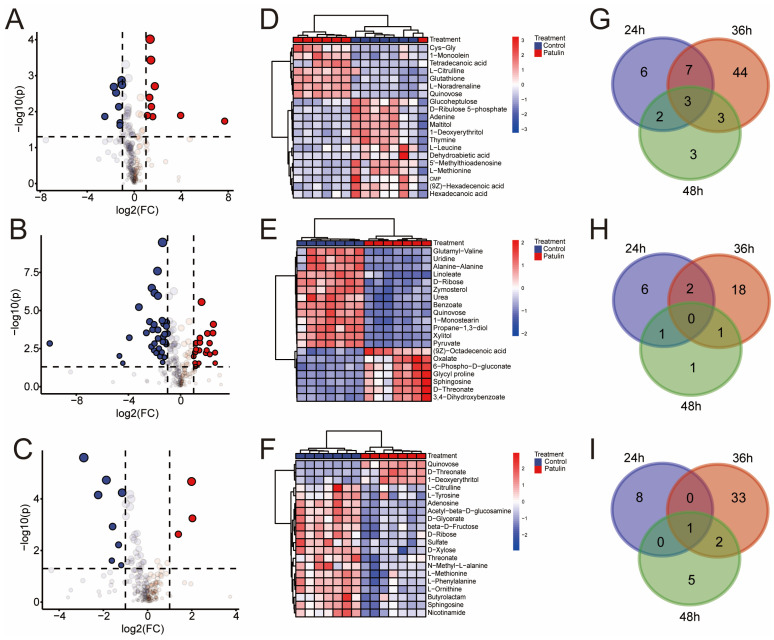
Subfigures (**A**–**C**) are the volcano plots of control group and patulin treatment groups at different degradation times, for which the up-regulated (red), down-regulated (blue), and non-regulated (grey) groups were determined based on the fold change ≥2 or fold change ≤0.5, employing a *p*-value < 0.05: (**A**) 24 h; (**B**) 36 h; (**C**) 48 h. (**D**–**F**) are the heatmaps of control group and patulin treatment groups at different degradation times, wherein the top twenty different metabolites with a VIP-value > 1 were selected for display: (**D**) 24 h; (**E**) 36 h; (**F**) 48 h. (**G**–**I**) are Venn diagrams depicting the shared and specific metabolites between the different groups of *A. niger* FS10: (**G**) total accumulated metabolites; (**H**) up-accumulated metabolites; (**I**) down-accumulated metabolites.

Figure 4A–C and Tables S1–S3 show the results concerning 24 h_patulin vs. 24 h_control, 36 h_patulin vs. 36 h_control, and 48 h_patulin vs. 48 h_control, which were determined based on the fold change ≥2 or fold change ≤0.5, with a *p*-value < 0.05. The results showed that 18, 57, and 11 differentially accumulated metabolites were identified for the 24 h_patulin vs. 24 h_control, 36 h_patulin vs. 36 h_control, and 48 h_patulin vs. 48 h_control tests, respectively (Figure 4G). The quantities of significantly upregulated metabolites were 9 (24 h), 21 (36 h), and 3 (48 h) (Figure 4H), and the quantities of significantly downregulated metabolites were 9 (24 h), 36 (36 h), and 8 (48 h) (Figure 4I). Notably, the number of differentially accumulated metabolites at the 36 h time point was significantly higher than the quantities of metabolites at the 24 h and 48 h time points. The clustering heatmap provides a visualization of the data. Each color block represents the metabolite level in the sample, each row represents the type of metabolite, and each column represents the sample. In Figure 4D–F, the variables’ importance in projection ≥1 for the first 20 different metabolites is presented. Under different processing times, all the parallels of each sample can be grouped together, and the metabolite abundances of the control group and the patulin treatment group are relatively different. Obviously, these findings are suitable for the next step of the metabolic pathway enrichment study, in which the corresponding changes in the metabolism of *A. niger* FS10 during patulin degradation are further discussed.

**Figure 5:**
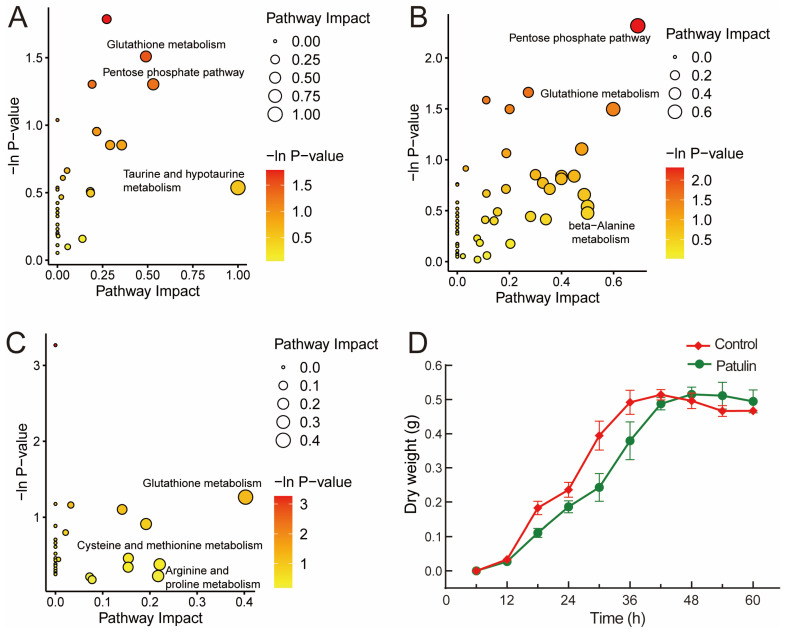
(**A**–**C**) constitute the pathway analysis of the identified metabolites affected in the patulin treatment groups. Significantly changed pathways based on enrichment and topology analysis are shown. The *x*-axis represents the pathway impact, and the *y*-axis represents pathway enrichment. The timepoints of the pathway enrichment analysis of *A. niger* FS10’s metabolites are as follows: (**A**) 24 h; (**B**) 36 h; (**C**) 48 h. (**D**) shows the growth curve of *A. niger* FS10. The red curve represents the control group, while green curve represents patulin treatment group.

MetaboAnalyst 5.0 was used to analyze the pathway mapping of the *p*-value < 0.05 metabolites (Figure 5A–C). Among the three groups, the 48 h groups had the lowest pathway impact values; meanwhile, the pathway impact values were higher in the 24 h and 36 h groups. This finding was not unexpected and was consistent with the previous PCA and volcano plots analyses. The growth curve of *A. niger* FS10 (Figure 5D) shows that at the 24 h time point, both the control group and the patulin treatment group *A. niger* FS10 are in the growth phase. At the 36 h time point, *A. niger* FS10 in both the control groups stabilized, whereas the patulin treatment group was in the logarithmic growth phase. Both groups reached a stable phase at the 48 h time point. These results were in good agreement with the metabolic response of *A. niger* FS10. Moreover, significant effects on glutathione metabolism were observed in the three groups, but the effect of the 48 h group were reduced compared to the effects of the 24 h and 36 h groups. Notably, the pentose phosphate pathway in the three groups was significantly affected at both the 24 h and 36 h time points but not at the 48 h time point. This finding suggests that glutathione metabolism and the pentose phosphate pathway may be related to the stress response of *A. niger* FS10 to patulin.

### 3.5. Changes in Patulin Concentration and Nutrients during Apple Pomace Fermentation

**Figure 6:**
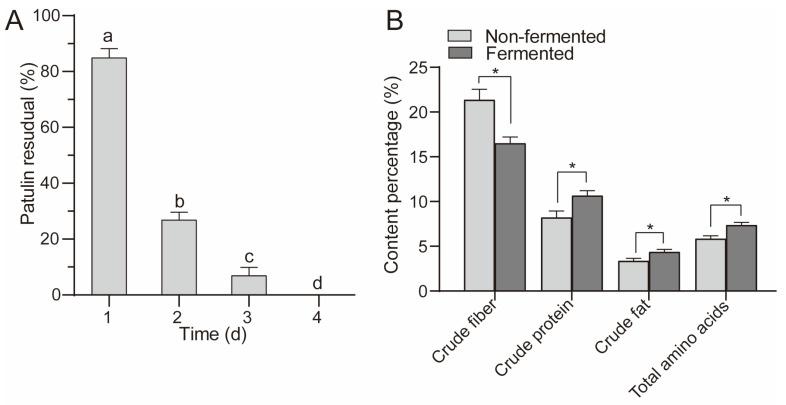
(**A**) shows residual rate of patulin in apple pomace feed. (**B**) shows the changes in the nutrients in the fermented and non-fermented apple pomace feed. Different letters indicate a significant difference between them (*p* < 0.05); “*” means *p* < 0.05.

As shown in Figure 6A, with the extension of the fermentation time, the residual amount of patulin gradually decreased. The residual amount of patulin after fermentation for 2 d was 26.89%, while that of patulin after fermentation for 4 d was not detected, indicating that the patulin in apple pomace was completely degraded by *A. niger* FS10. The concentrations of crude cellulose, crude protein, crude fat, and total amino acids in the apple pomace after 4 d of fermentation were determined. Compared with non-fermentation, the crude fiber content in the apple pomace after fermentation was significantly reduced, while the crude protein, crude fat, and total amino acid content increased significantly (Figure 6B), indicating that *A. niger* FS10 can ferment the cellulose in apple pomace into nutrients such as protein and amino acids.

## 4. Discussion

Biological detoxification refers to the process of using microorganisms or enzymes produced by metabolism to act on toxins and destroy their toxic groups, thereby generating non-toxic or low-toxicity degradation products [23]. This method not only avoids significantly reducing the nutritional value of food but also meets food industry requirements [24]. Numerous microorganisms have been shown to effectively remove patulin. In the current study, when the food-safe strain *A. niger* FS10 was cultured with 2 μg/mL of patulin, the concentration of patulin was significantly reduced and disappeared after 40 h (Figure 1A). Compared with the control group, the culture filtrate broth showed a small degree of patulin degradation at all tested time points; however, the efficiency of this degradation was inferior and did not change with time (Figure 1B). This result suggests that the *A. niger* FS10 culture filtrate was not the main degradational agent towards patulin. Fungi remove patulin via two mechanisms: adsorption and biodegradation [25]. The heat-treated mycelia of *A. niger* FS10 could not reduce the patulin concentration, unlike the unheated mycelia, which significantly decreased the patulin concentration (Figure 1C). When incubated, the patulin concentration also decreased with an increase in fungal density (Figure 1D). These results indicate that *A. niger* FS10 mainly reduced the level of patulin in the environment via biodegradation in living cells. These results are consistent with the findings of Zhu et al. [26].

Before the biological control offered by mycotoxin contamination technology is applied, the degradation products must be identified and their safety must be evaluated. Two main degradation products of patulin have been reported: desoxypatulinuc acid (DPA) and ascladiol. *Rhodotorula mucilaginosa* converted patulin to DPA [27], whereas *Candida guilliermondii* converted patulin to ascladiol [28]. The retention time of the ascladiol peak in the HPLC chromatogram occurred earlier than that of the patulin peak, and the peak time of DPA occurred after the peak time of patulin [22]. In the current study, after *A. niger* FS10 was cultured with patulin, only a new peak with a retention time of 4.31 min was shown before patulin degradation. The degradation product was identified by Q Exactive Focus analysis; consequently, its determined molecular formula was C_7_H_8_O_4_. The data acquired via MS/MS spectra indicated that the main degradation product was ascladiol. Therefore, *A. niger* FS10 was able to convert patulin to ascladiol.

The toxic effects of patulin on the liver have been confirmed in many studies [29]. The human liver cancer cell HepG2 is commonly used as a liver toxicology model for toxicity evaluation research [30]. The presence of patulin can induce oxidative stress-mediated apoptosis in cells [31]. In the present study, the human liver cancer cell HepG2 was used to evaluate the toxicity of degradation products. The results of the study indicated that when patulin was applied to HepG2 cells, the apoptosis level was significantly higher than that of the control group, and the cell survival rate was significantly lower. However, after treatment with *A. niger* FS10, the degraded products could significantly improve the survival rate of cells. The apoptosis level was then significantly lower than that of the patulin group, indicating that the toxicity of patulin was significantly reduced after treatment with *A. niger* FS10.

In our metabolomics analysis, after patulin stimulated the FS10 strain, it was observed that the *A. niger* FS10 metabolites were enriched in various metabolic pathways, indicating the complexity of patulin degradation by *A. niger* FS10. The pentose phosphate pathway was significantly affected at the 24 h and 36 h time points but not at the 48 h time point. Glutathione metabolism in the three time groups exerted a significant effect, but the effect of the 48 h group was less than that of either the 24 h group or the 36 h group. The pentose phosphate pathway plays a key role in maintaining the normal growth of cells and in DNA damage repair [32]. Studies have reported that patulin treatment can induce DNA damage, including the inhibition of DNA repair and replication [33], and the pentose phosphate pathway can help improve the ability to repair DNA damage [34]. Glutathione is considered a scavenger of patulin [35]. Glutathione and patulin can form sulfhydryl-coupling compounds, which can lead to a sharp decrease in the content of glutathione in the cell and weaken its ability to scavenge oxygen free radicals, thus causing oxidative damage to cells [36]. However, in this study, the glutathione content after patulin treatment for 24 h was significantly increased relative to that of the control group. This finding indicates that *A. niger* FS10 produces more glutathione after patulin stress (Figure S4), thereby reducing the oxidative stress damage attributed to patulin and improving the tolerance of *A. niger* FS10 to patulin.

Apple pomace contains high moisture content, is rich in nutrients, and is easily infected by harmful microorganisms, and these properties lead to patulin contamination. Many studies have reported regarding the microbial fermentation of apple pomace. *A. niger* van. Tieghem MTCC 281 can use apple pomace as a substrate for fermentation in order to produce citric acid [37]. Berovic et al. [38] developed a method for using *A. niger* to produce pectinase via the solid-state biological fermentation of apple pomace. Joshi et al. [39] fermented apple pomace in a solid state to convert it into an animal feed with high nutritional value. However, few studies have focused on patulin contamination in apple pomace. Our research shows that *A. niger* FS10 fermented patulin-contaminated apple pomace for 4 d, that patulin in the apple pomace could be completely degraded, and that the concentrations of protein, fat, amino acids, and other nutrients in the apple pomace were significantly improved following fermentation. This research provides new ideas with respect to the utilization of apple pomace.

## 5. Conclusions

The results of this study show that *A. niger* FS10 can completely degrade patulin and convert it into the diol ascladiol. This degradation method can effectively reduce the toxic damage to cells effected by patulin. *A. niger* FS10 can improve the ability to repair the damage caused by patulin stress via the pentose phosphate pathway and the glutathione pathway and increase patulin tolerance by producing more glutathione. Using *A. niger* FS10 to ferment patulin-contaminated apple pomace not only completely degrades the patulin in the apple pomace after fermentation but also significantly improves its nutritional value. This study proposes a new method for patulin degradation and provides a theoretical basis for its application. However, the degradation mechanism underlying the ability of *A. niger* FS10 to degrade patulin has yet to be fully established. In the future, studies should cultivate *A. niger* FS10 such that it can be more suitable for the degradation environment of complex samples. Meanwhile, the degradational mechanism of mycotoxins effected by *A. niger* FS10 was investigated in order to ascertain the relevant degradation enzymes, which may lead to the commercialized production of these enzymes through synthetic biological technology.

## Data Availability

Data is contained within the article.

## Supplementary Materials

The following supporting information can be downloaded at: https://www.mdpi.com/article/10.3390/foods12020382/s1, Figure S1: PCA of control samples (blue) and patulin treatment samples (red) in different degradation time. (a) 24 h, Variance of PC1 is 46.9% and variance of PC2 is 15.6%. (b) 36 h, Variance of PC1 is 40.9% and variance of PC2 is 21.9%. (c) 48 h, Variance of PC1 is 36% and variance of PC2 is 16.3%. Results are expressed as means of seven replicates; Figure S2: Partial Least Squares Discrimination Analysis (PLS-DA) (a) 24 h. (b) 36 h. (c) 48 h. The control group (blue), the patulin treatment group (red). There was a significant separation between each group, indicating that there was a significant difference in the metabolism of *A. niger* FS10 between the control group and the patulin treatment group. Figure S3: Permutation test (A) 24 h, R2Y = 0.969, Q2Y = 0.933. (B) 36 h, R2Y = 0.981, Q2Y = 0.946. (C) 48 h, R2Y = 0.937, Q2Y = 0.798. The scores of R2Y and Q2Y in each group were higher, which indicated that the prediction of the model was reliable and could be used for the analysis of differential metabolites; Figure S4: Boxplots of the relative concentration change of glutathione. (A) 24 h. (B) 36 h. (C) 48 h. The control group (blue), the patulin treatment group (red); Table S1: Results of metabolomics analysis after 24 h treatment with patulin; Table S2: Results of metabolomics analysis after 36 h treatment with patulin; Table S3: Results of metabolomics analysis after 48 h treatment with patulin
