# Supplementary material for: Efficient Biodegradation of Patulin by Aspergillus niger FS10 and Metabolic Response of Degrading Strain
Source: Foods. 2023 Jan 13;12(2):382. doi: 10.3390/foods12020382 (PMC9858360; doi:10.3390/foods12020382)
Supplement: Supplementary file 1 [file foods-12-00382-s001.zip › Supplementary materials.pdf]

<u>Supplementary Information</u>

Efficient Biodegradation of Patulin by *Aspergillus niger* FS10 and Metabolic Response of Degrading Strain

Yang Yang [1,†], Jian Ji [1,2,†], Shang Wu [1], Yongli Ye [1], Lina Sheng [1], Yinzhi Zhang [1] and Xiulan Sun [1,*]

[1] School of Food Science, State Key Laboratory of Food Science and Technology, National Engineering Research Center for Functional Foods, School of Food Science Synergetic Innovation Center of Food Safety and Nutrition, Joint International Research Laboratory on Food Safety, Jiangnan University, Wuxi 214122, China
[2] College of Food Science and Pharmacy, Xinjiang Agricultural University, Ürümqi 830052, China
* Correspondence: sxlzzz@jiangnan.edu.cn; Tel.: +86-510-85329015; Fax: +86-85328726
† These authors contributed equally to this work.

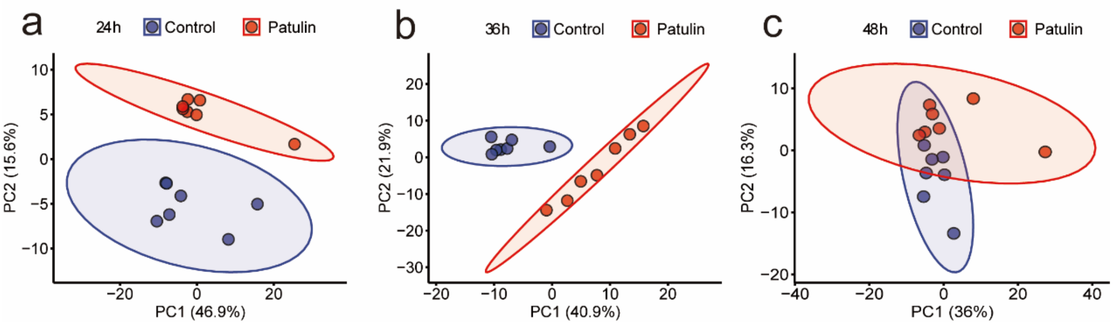

Figure S1 PCA of control samples (blue) and patulin treatment samples (red) in different degradation time. (a) 24 h, Variance of PC_1 is 46.9% and variance of PC_2 is 15.6%. (b) 36 h, Variance of PC_1 is 40.9% and variance of PC_2 is 21.9%. (c) 48 h, Variance of PC_1 is 36% and variance of PC_2 is 16.3%. Results are expressed as means of seven replicates.

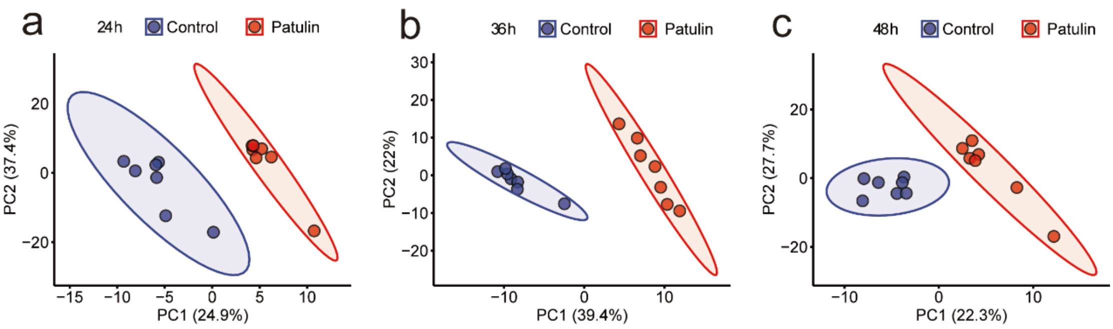

Figure S2 Partial Least Squares Discrimination Analysis（PLS-DA）(a) 24 h. (b) 36 h. (c) 48 h. The control group (blue), the patulin treatment group (red). There was a significant separation between each group, indicating that there was a significant difference in the metabolism of A. niger FS10 between the control group and the patulin treatment group.

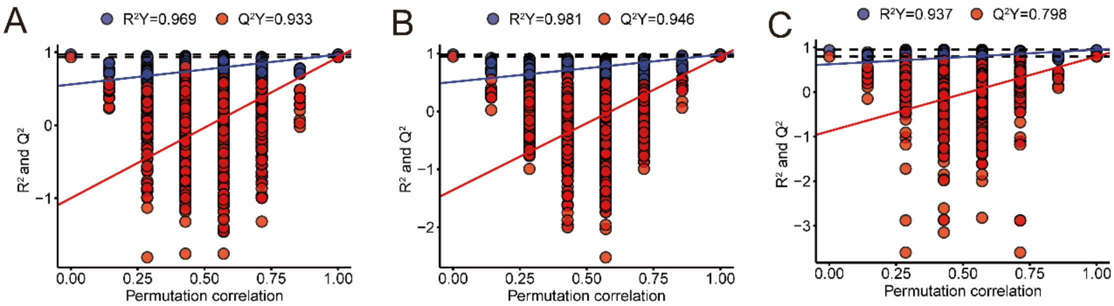

Figure S3 Permutation test (A) 24 h, R^2Y=0.969, Q^2Y=0.933. (B) 36 h, R^2Y=0.981, Q^2Y=0.946. (C) 48 h, R^2Y=0.937, Q^2Y=0.798. The scores of R^2Y and Q^2Y in each group were higher, which indicated that the prediction of the model was reliable and could be used for the analysis of differential metabolites.

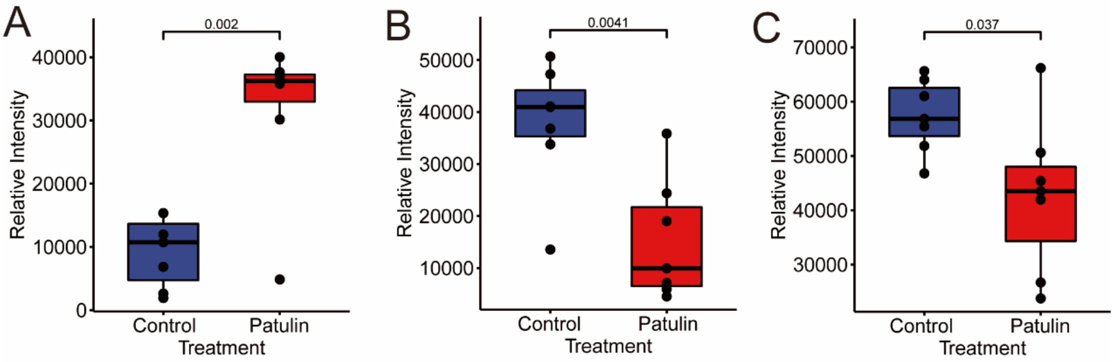

Figure S4 Boxplots of the relative concentration change of glutathione. (A) 24 h. (B) 36 h. (C) 48 h. The control group (blue), the patulin treatment group (red)
